# In-vitro and in-vivo assessment of nirmatrelvir penetration into CSF, central nervous system cells, tissues, and peripheral blood mononuclear cells

**DOI:** 10.1038/s41598-024-60935-5

**Published:** 2024-05-10

**Authors:** Sean N. Avedissian, Johid R. Malik, Anthony T. Podany, Michael Neely, Nathaniel J. Rhodes, Kimberly K. Scarsi, Marc H. Scheetz, Michael J. Duryee, Ukamaka O. Modebelu, Timothy M. Mykris, Lee C. Winchester, Siddappa N. Byrareddy, Courtney V. Fletcher

**Affiliations:** 1 Antiviral Pharmacology Laboratory, College of Pharmacy, University of Nebraska Medical Center, 986145 Nebraska Medical Center, Omaha, NE 68198-6145 USA; 2 Department of Pediatrics, Division of Infectious Diseases, University of Southern California, Children’s Hospital Los Angeles, Los Angeles, CA USA; 3 Department of Pharmacy Practice, Chicago College of Pharmacy, Midwestern University, Downers Grove, IL USA; 4 Pharmacometrics Center of Excellence, Midwestern University, Downers Grove, IL USA; 5 Division of Infectious Diseases, Department of Medicine, University of Nebraska Medical Center, Omaha, NE USA; 6 Division of Rheumatology, Department of Pharmacology & Experimental Neurosciences Internal Medicine, University of Nebraska Medical Center, Omaha, NE USA; 7 Department of Pharmacology & Experimental Neurosciences, University of Nebraska Medical Center, Omaha, NE USA

**Keywords:** Nirmatrelvir/ritonavir, Blood–brain-barrier, Central nervous system, Viral infection, Clinical pharmacology, Pharmacodynamics, Pharmacokinetics

## Abstract

Three years after SARS-CoV-2 emerged as a global infectious threat, the virus has become endemic. The neurological complications such as depression, anxiety, and other CNS complications after COVID-19 disease are increasing. The brain, and CSF have been shown as viral reservoirs for SARS-CoV-2, yielding a potential hypothesis for CNS effects. Thus, we investigated the CNS pharmacology of orally dosed nirmatrelvir/ritonavir (NMR/RTV). Using both an in vitro and an in vivo rodent model, we investigated CNS penetration and potential pharmacodynamic activity of NMR. Through pharmacokinetic modeling, we estimated the median CSF penetration of NMR to be low at 18.11% of plasma with very low accumulation in rodent brain tissue. Based on the multiples of the 90% maximal effective concentration (EC_90_) for SARS-CoV-2, NMR concentrations in the CSF and brain do not achieve an exposure level similar to that of plasma. A median of only 16% of all the predicted CSF concentrations in rats were > 3xEC_90_ (unadjusted for protein binding). This may have implications for viral persistence and neurologic post-acute sequelae of COVID-19 if increased NMR penetration in the CNS leads to decreased CNS viral loads and decreased CNS inflammation.

## Introduction

Global cases of coronavirus infectious disease 2019 (COVID-19) continue to rise daily^1,2^. Although SARS-CoV-2 is often referred to as a respiratory virus, in addition to the lung it has been found in tissues including the brain, liver, intestine, feces, heart, and kidneys of individuals with COVID-19^3^. Moreover, COVID-19 has been demonstrated to infect mononuclear cells. In postmortem lung T-cells, the presence of COVID-19 antigen was observed in  CD4 positive T-cells indicating SARs-CoV-2 infection, and there have been reports of antibody-mediated infection in monocytes and macrophages as well^4–6^.The impact of COVID-19 on human health has led to significant investment in new strategies including the development of new therapeutic agents to reduce the risk of infection, disease, and negative outcomes.

One available oral antiviral treatment for COVID-19 is nirmatrelvir/ritonavir (NMR/RTV; PAXLOVID™)^7^. This drug is a combination of a SARS-CoV-2 non-structural protein 5(NSP5) protease inhibitor (PI) NMR, and RTV (weak-PI) used in a low-dose as a pharmacokinetic (PK) enhancer to increase the concentrations of NMR in the blood via inhibition of hepatic oxidative metabolism^8^. NMR is a peptidomimetic inhibitor of the SARS-CoV-2 main protease (Mpro), also referred to as 3-chymotrypsin-like protease (3CLpro) or NSP5 protease. Inhibition of SARS-CoV-2 Mpro renders the virus incapable of processing the polyproteins pp1a and pp1ab, preventing replication^9^. NMR/RTV received FDA approval on May 25th 2023, as the first oral antiviral treatment for mild to moderate COVID-19 in adults who are at high risk for severe COVID-19^10^. Currently this combination’s only utility is against SARs-CoV-2 infection.

Neurological complications associated with SARS-CoV-2 infection are not well understood. Post-acute sequelae of COVID-19 (PASC), also known as Long COVID, is a chronic syndrome that affects some individuals who have recovered from acute COVID-19 illness^11^. Based on available literature, the related incidence, risk factors, possible pathophysiology, and proposed management of neurological manifestations has been summarized by Moghimi and colleagues^12^. While the majority of SARs-CoV-2 infected persons no longer show symptoms after recovering from infection, some experience persistent neuro-specific PASC (neuroPASC) symptoms (e.g., depression, anxiety, difficulty in concentrating, and central nervous system [CNS] disturbances)^13^ lasting months or even years after the infection^14,15^. Interestingly, fatigue has been observed as one of the most common symptoms associated with Long COVID^16,17^. The etiology of neuroPASC is unclear, and the exact mechanisms of SARS-CoV-2 entry into the CNS are uncertain. Some theories for entry include infection of the endothelium, access through the blood–brain barrier (BBB), and through nervous tissue conduits that bypass the BBB. Given that cells in the CNS can be infected with SARS-CoV2^18^, it is plausible that CNS infections lead to the neurological complications described by neuroPASC^19–21^. Mechanisms for SARS-CoV-2 associated neurological complications are still currently being explored^22^. Another theory is that neuroPASC is due to prolonged inflammation present in the CNS post-infection. This theory is supported by both clinical and animal data in persistent SARs-CoV-2 infection^23,24^. Clinical data from autopsy sampling performed on the CNS of patients who died from COVID-19 found viral RNA, with patients having detectable CNS virus from 4–230 days after infection^25^. A study by Beckman and colleagues showed that COVID neuroinvasion (non-human primate model) was more significant and widespread throughout the olfactory cortex in older animals than younger ones. They also found axonal spread of the virus from the nasal olfactory epithelium. In the older monkeys, there was an increase in viral load, more pronounced cellular alterations, and neuroinflammation^26^. Given data to support viral entry into the CNS^27^, and the known neurological issues associated with neuroPASC, early and effective antiviral treatment of acute COVID-19 may offer hope in preventing or reducing neuroPASC occurrence and severity^28,29^.

Currently, there are no published data on NMR concentrations in the CNS when given orally. It is unknown if NMR can cross the BBB or the blood-cerebrospinal fluid (CSF) barrier (BCSFB)  and achieve therapeutic concentrations necessary to treat SARS-CoV-2 infection in the CNS. Given the limited treatment options available for COVID, it is essential to evaluate whether current treatment can be maximized to ensure viral eradication. Treatment and prevention of neuroPASC caused by virus in the CNS would require therapeutic CNS NMR concentrations, which are a function of effective concentration goals (EC_50–90_), brain penetration and dose. Suboptimal drug concentrations in the CNS during acute treatment may unintentionally contribute to neuroPASC. A general principle for treatment of infectious diseases is the need for adequate drug concentration at the site of infection^30^. CNS penetration is dependent on many factors that control the ability and amount of a drug that can cross the BBB (e.g., lipophilicity, molecular weight, molecular charge, etc.). Thus, to reach effective drug concentrations in the CNS, strategies to raise the systemic drug levels by increasing dose, frequency or duration, or changing formulation or route of administration, may be necessary^31^. However, increasing the drug dose may significantly increase the risk of systemic toxicity. Preclinical studies investigating penetration into reservoirs are necessary to determine if therapeutic concentrations are clinically achievable. Several clinical trials are currently ongoing looking at using NMR/RTV as a treatment strategy for patients that are highly symptomatic with Long COVID. One ongoing trial is using NMR/RTV for 15-days at the current dose to see if this treatment will provide relief in those suffering with Long COVID (NCT05576662; NCT0559369; NCT05668091). These efforts support the need to assess CNS penetration of NMR/RTV. With this premise, the critical and initial step is to understand NMR/RTV penetration utilizing pre-clinical models. Accordingly, the objective of this study was to use in vitro and in vivo preclinical models to determine NMR penetration into the CNS. Astrocytes and pericytes are integral to BBB structure controlling the drug penetration across BBB and uptake of the chemotherapeutic agents for CNS entry. Animal models provide a way to probe questions that require invasive sampling clinically. Our approach was to use an in vitro system consisting of cells of the BBB to explore the ability of NMR/RTV to enter these cells, and in vivo measurements of NMR/RTV in CSF and other anatomical sites utilizing a rat model.

## Results

### In-vitro drug uptake

**Figure 1:**
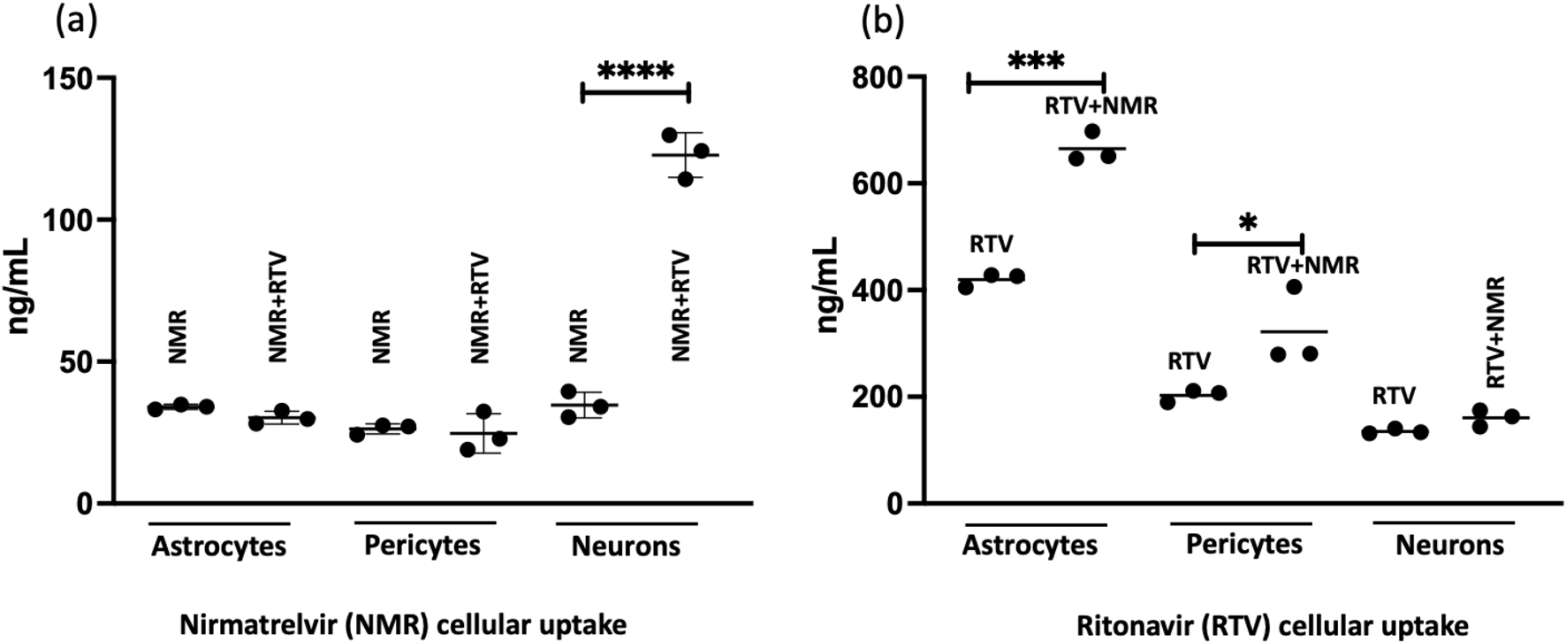
In-vitro analysis of NMR and RTV penetration into three different human brain cells. (**a**) Evaluation of NMR uptake by cells in the absence or presence of RTV and (**b**) intracellular RTV uptake in the absence or presence of NMR. The p-values (*) indicate, * =  < 0.05, *** =  < 0.0002 and **** =  < 0.0001. *NMR* nirmatrelvir, *RTV* ritonavir.

The mean ± SD uptake of NMR alone compared with in the presence of RTV by neurons was 34.7 ng/mL ± 0.88 and 122.8 ± 7.8 ng/mL, respectively (P < 0.0001). The mean ± SD of RTV uptake in astrocytes and pericytes in the presence or absence of NMR was 419.7 ng/mL ± 12.8 ng/mL vs. 665.2 ng/mL ± 28.3 ng/mL for astrocytes (P < 0.0002) and 202.6 ng/mL ± 11.5 ng/mL vs 321.9 ng/mL ± 72.6 ng/mL vs. for pericytes (P < 0.05), respectively. Overall, the maximum NMR uptake was low (5.5%; i.e., 2200 ng/mL administered vs. 122.8 ng/mL uptake: 122.8/2200 = 5.5%; Fig. 1a: neurons), as seen with neurons in the presence of RTV. The uptake for NMR increased > 3.6-fold in neurons in the presence of RTV (34.7 ng/mL to 122.8 ng/mL). We observed low (< 2%) uptake of NMR by astrocytes or pericytes (Fig. 1a: astrocytes, pericytes) in the presence or absence of RTV. Further, we observed moderate (42%) uptake of RTV in astrocytes (Fig. 1b: astrocytes, 1000 ng/mL administered vs. 419.7 ng/mL uptake), and in the presence of NMR, RTV uptake significantly increased to 66.5% (1000 ng/mL administered vs. 665.2 ng/mL uptake).

### Characteristics of animal cohort

A total of 10 rats received NMR/RTV orally by gavage and had plasma and CSF concentrations obtained throughout dosing and tissue samples collected at completion. Each day, rats had an average of 6.5 plasma concentrations and 1.8 CSF concentrations sampled over the 5-day protocol (Total: 327 plasma, 83 CSF concentrations). One animal had intracisternal catheter failure before the collection of any CSF samples.

### NMR PK model and parameter estimates

The final PK model was a three-compartment first-order oral absorption model with a bioavailability (F) covariate (Supplemental Fig. 1), AIC = 771.4 (Supplemental Table 1). The final model’s median PK parameter values are given in Table 1. The PK model was fit-for-purpose with low bias in both plasma and CSF (− 0.0778 mg/L and − 0.0263 mg/L). Bayesian predictions from the final model explained the variation in the observed individual animal concentrations well (r^2^ = 0.76 and 0.51 for plasma and CSF, respectively [Supplemental Fig. 2]).

**Table 1:** Median parameter values from final model (a) and individual animal NMR half-life and average bioavailability (b).

(a)				
PK parameter	Median	CV%	Variance	Shrink%^
Ka (hr-1)	0.51	47.17	0.1	2.37
CL (L/hr)	0.23	49.98	0.02	0.58
K_23_ (hr-1)	0.05	105.93	0.05	1.13
$K_30_ (hr-1)	0.24	43.73	0.01	11.96
Vc (L)	1.05	41.12	0.15	0.78
Vcsf (L)	3.46	63.98	6.49	5.56

(b)				
Rat	Half-life (h)	Average relative bioavailability (F)*	Average T_max_ (h)	
1	1.87	0.58	2.17	
2	3.86	0.58	3.63	
3	2.46	0.46	2.25	
4	1.32	0.55	1.56	
5	3.23	0.54	1.22	
6	1.46	0.48	1.65	
7**	3.23	0.58	1.19	
8	2.80	0.32	1.7	
9	0.98	0.62	1.15	
10	2.65	0.41	1.9	
Median (IQR)	2.55 (1.43–3.23)	(0.45–0.58)	1.675 (1.21–2.19)	
Mean (SD)^#^			1.84 (0.73)	

*PK* pharmacokinetic, *CV%* coefficient of variation percent, *CL* NMR clearance, *V*_*c*_ volume central compartment, *V*_*csf*_ volume cerebrospinal fluid compartment, *K*_*23*_ rate constant to cerebrospinal fluid from central compartment, *K*_*30*_ elimination rate constant from CSF compartment, *IQR* interquartile range, T_max_ time at which C_max_ was first observed.

*Bioavailability was estimated after each dose given the variability of oral absorption, as described in Methods.

**Rat 7 only completed 1 day of treatment.

^#^Calculated to compare to literature values.

^^^Estimation to assess if the data are insufficient to precisely estimate the individual parameters.

^$^Estimation denotes overall elimination of NMR from the CSF, including uptake by various types of cells in the CNS.

### NMR PK exposures and percent (%) CSF penetration

**Figure 2:**
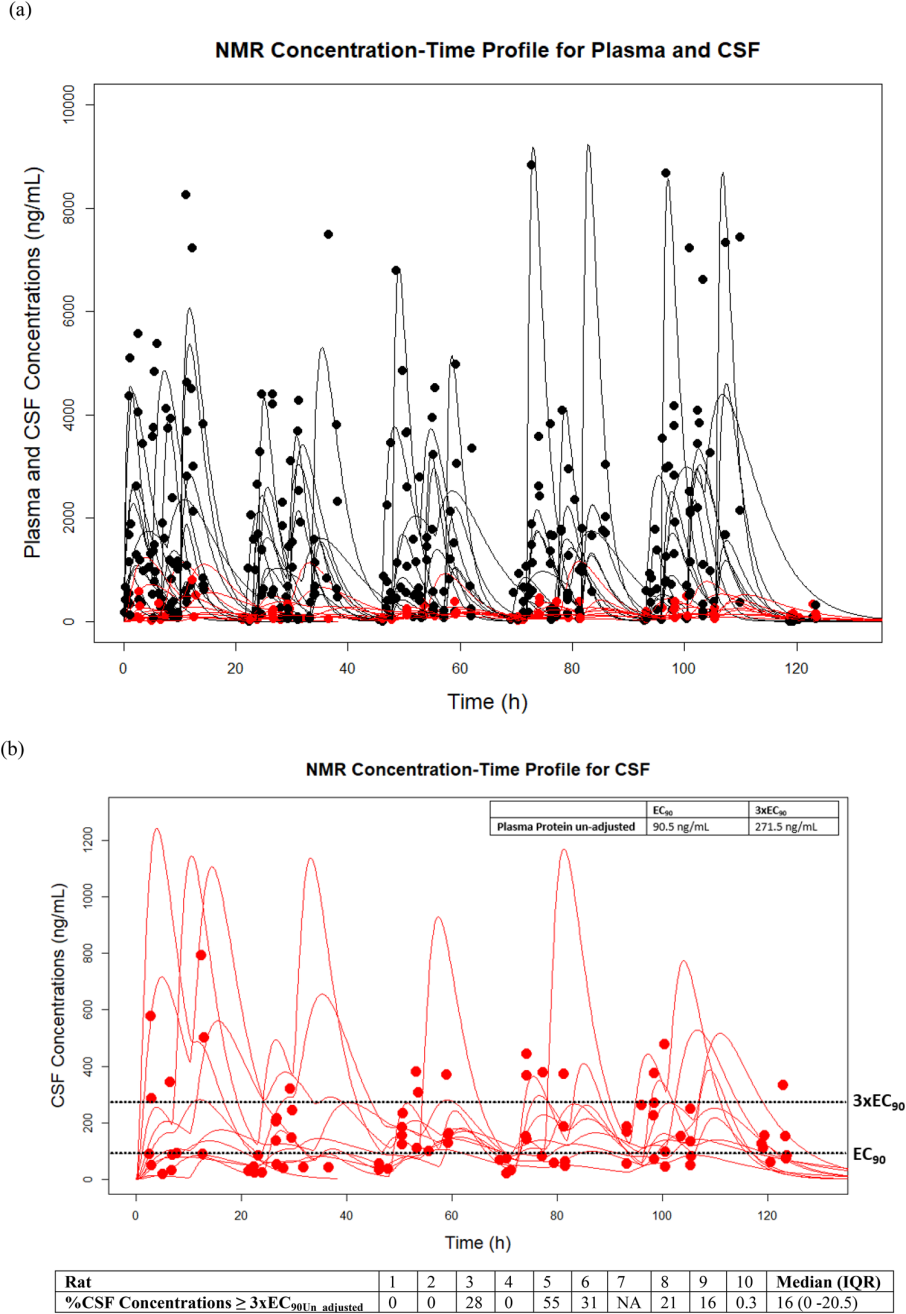
Plasma ((**a**) black) and CSF ((**b**) red) observed versus Bayesian predicted plots for all animals compared to EC_90Un_adjusted_ values (dotted black line). The black and red lines represent the predictions where the filled circles represent the observed collected concentrations. A median of 16% of all the predicted CSF concentrations in rats were > 3xEC_90Un_adjusted_. *Units on plot converted to ng/mL for consistency. *NMR* nirmatrelvir, *CSF* cerebrospinal fluid, *EC*_*90*_ 90% maximal effective concentration (unadjusted for protein binding given CSF, *90.5 ng/mL* 0.0905 mg/L and 271.5 ng/mL = 0.2715 mg/L).

The overall PK exposures for all rats are summarized in Table 2. The median (IQR) NMR penetration into the CSF was low at 18.1% (7.65–30.59) (calculated from highest predicted concentration [C_max_]) and 15.2% (7.55–29.92) (calculated from area under the concentration–time curve [AUC]). The complete list of NMR penetration into CSF for each animal can be found in Table 2. Further, observed versus Bayesian predicted concentration time profiles for plasma and CSF vs. 90% maximal effective concentration [EC_90_] and 3xEC_90_ values can be found in Fig. 2. The CSF Bayesian prediction concentration time profiles for all animals showed the median (IQR) percent of time CSF concentrations were ≥ 3xEC_90_ unadjusted for plasma protein binding (EC_90Un_adjusted,_ note: adjusted = EC_90Adjusted_) was 16% (0–20.5) (Fig. 2b).

**Table 2:** NMR plasma and CSF PK exposures estimated using Bayesian posteriors for AUC_0-endoftreatment_ and C_max___0-5 days_ and percent penetration of NMR into the CSF compared to blood.

Animal	C_max_0-5 days_ (ng/mL) Plasma	AUC_0-endoftreatment_ (µg*hr/mL) Plasma	AUC_daily_average_ (µg*hr/mL) Plasma	C_max_0-5 days_ (ng/mL) CSF	AUC_0-endoftreatment_ (µg*hr/mL) CSF	AUC_daily_average_ (µg*hr/mL) CSF	% Penetration by C_max_ CSF/Plasma	% Penetration by AUC CSF/Plasma
1	2270	160	32	105.81	7.11	1.42	4.66	4.44
2	3660	189	37.8	252	12.5	2.5	6.89	6.61
3	2020	92.7	18.54	655.8	28.9	5.78	32.47	31.175
4	796	76.3	15.26	144	11.6	2.32	18.10	15.20
5	1860	80.7	16.14	1169.9	52.21	10.44	62.84	64.7
6	2218	128	25.6	560	30.2	6.04	25.27	23.59
7^#^	4550	19.55	3.91	NA	NA	NA	NA	NA
8	3857.9	99.8	19.96	1107	28.6	5.72	28.70	28.66
9	4879.3	205	41	410	17.4	3.48	8.41	8.49
10	1862.7	128	25.6	271.6	17.6	3.52	14.58	13.75
Median (IQR)	2240 (1860–4030)	113.9 (79.6–167.3)	22.78 (15.92–33.45)	410 (200–880)	17.6 (12.05–29.55)	3.52 (2.41–5.91)	18.1 (7.65–30.59)	15.2 (7.55–29.92)
Median (IQ R)*	2220 (1860–3760)	128 (86.7–174.5)	25.6 (17.34–34.9)	–	–	–	–	–
Geometric mean*^#^ (Geometric SD factor)	2480 (1730)	–	20.25 (1.956)	–	–	–	–	–

Units for C_max_ converted to ng/mL for consistency. AUC kept in µg*hr/mL.

*C*_*max*_ maximum concentration, *AUC* area under the curve, *CSF* cerebral spinal fluid, *T ½* half = life, *IQR* interquartile range**,** *SD* standard deviation.

*Excluding rat 7 as no CSF was obtained from this animal, #Calculated to compare to clinical data.

### Tissue and peripheral blood mononuclear cells (PBMC) NMR concentrations

**Figure 3:**
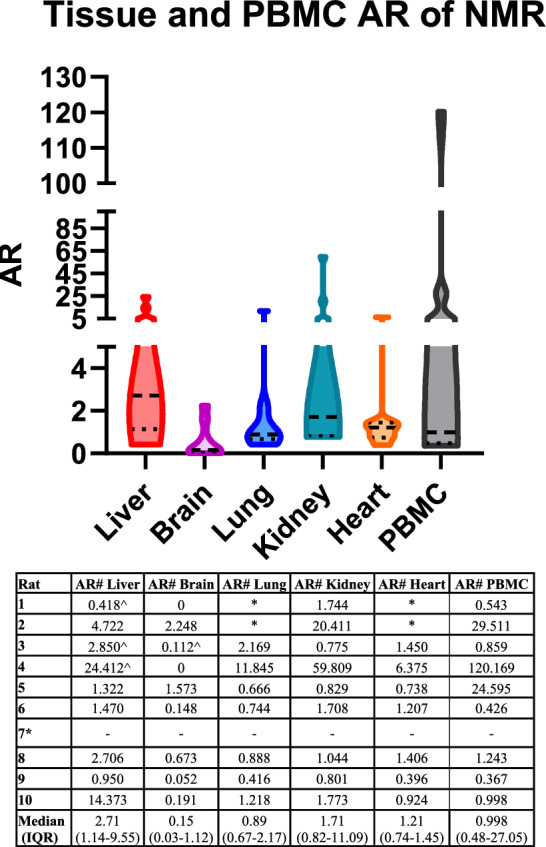
Violin plots of tissue and PBMC AR for NMR. The highest median NMR tissue AR was observed in the liver and kidney, while the lowest median NMR tissue AR was observed in brain tissue. *Rat 7 only completed 1 day of treatment, no PMBC levels available. Rats 1 and 2 do not have lung or heart NMR concentrations due to tissue processing complications. ^#^Calculated as a ratio of observed plasma NMR levels vs. tissue/PBMC levels at equivalent time of sampling. ^^^Calculated using plasma level predictions vs. observed concentrations due to plasma NMR levels being BLOQ. *AR* accumulation ratio, *BLOQ* below level of quantification, *IQR* interquartile range.

The overall tissue accumulation ratio (AR [desirable AR: > 1]) and tissue concentrations for NMR can be found in Fig. 3 and Supplemental Fig. 3. The highest median (IQR) NMR tissue ARs were observed in the liver (2.71 [1.14–9.55]), and kidney (1.71 [0.82–11.09]) while the lowest median NMR tissue AR was observed in brain tissue at 0.15 (0.03–1.12). Compared to all the tissues, the brain had the lowest median (23.83 ng/g, IQR: 10.94–46.85) NMR concentrations, which were all < 3xEC_90_ regardless of adjustment for protein binding. For PBMCs, the median (IQR) value for the cellular AR for NMR was 0.998 (0.48–27.05).

## Discussion

We found that NMR CSF concentrations in rats given oral NMR/RTV twice daily for five days were 15–18% of those in plasma, whether determined as a ratio of C_max_ or AUC (Table 2). Further, we found that tissue penetration of NMR in brain of the rats was low, which was consistent with the NMR cell uptake in our in vitro model. Saleh and colleagues used physiologically based pharmacokinetic (PBPK) modelling to predict whether NMR, remdesivir, and molnupiravir achieve effective concentrations against SARS-CoV-2 in human brain cells^32^. Their model predicted NMR concentrations exceeded the EC_90_ values in brain extracellular fluid concentrations, which is similar to what we found in rat CSF. However, they did not evaluate 3xEC_90_, or other multiplicative factors of the EC_90_ values, reflecting levels of plasma exposure observed clinically. Exposure–response relationships for SARS-CoV-2 viral loads relative to EC_90_ factors have not been evaluated in the CNS or other potential viral reservoirs. We utilized the concentration needed for 3xEC_90_ for SARS-CoV-2 as our pharmacodynamic (PD) target for the CSF, based on the FDA review from EPIC-HR showing 95% of participants had NMR trough concentrations ≥ 3 × EC_90_^33,34^. If the two EC_90_ values utilized in the PBPK simulation study by Saleh and colleagues for the Delta variant are multiplied by a factor of 3 (0.149 µM: ~ 100 ng/mL × 3 = 300 ng/mL), the majority of time is spent below this PD goal. In our study, we found that CSF concentrations of NMR aren’t maintained above the 3xEC_90Un_adjusted_ for SARS-CoV-2 (Fig. 2b**,** median overall CSF Concentrations ≥ 3 × EC_90_: 16%) for the entire dosing interval. As an exercise, we conducted Monte Carlo simulations (N = 1000, assuming 300 g rat, fraction unbound = 1) from the final rat population PK model to assess what doses (30–90 mg/kg) of NMR would be required to achieve the probability of target attainment of 50–100% time above different EC_90_ multiples (e.g., 0.5 − 3 × EC_90_) in the CSF. Based on the simulations, doses of > 90 mg/kg BID (Supplemental Figs. 4 and 5) in rats would be necessary to ensure all concentrations are > 3 × EC_90Un_adjusted_ (i.e., 271.5 ng/mL). Humanizing this dose based on allometric scaling would result in clinical doses of 900 mg of NMR BID (three times the current FDA approved dose) or potentially more frequent dosing of 300 mg every 4 h. The simulations did not account for varying the doses of RTV (which increase NMR concentrations) given the toxicity and drug-drug interactions of RTV that make it clinically difficult to justify pushing its dose higher^35^.

Our study is unique as we also looked at homogenized liver, brain, lung, kidney, and heart tissues, and PBMC concentrations of NMR in rats. As shown in Fig. 3 and Supplemental Fig. 3, brain tissues had the lowest concentrations and AR compared with other tissues. Only rat 7, which died within 4 h after the 2nd dose, had NMR concentrations > EC_90Un_adjusted,_ but no rat had NMR concentrations > 3 × EC_90_ regardless if comparing to adjusted or unadjusted for protein binding. For PBMCs, we found that intracellular NMR concentrations from PBMCs were detectable, and some rats had NMR PBMC concentrations above 3xEC_90Adjusted and Un_adjusted_. When compared to other PIs used in the treatment of HIV, the desirable human PBMC cellular AR is > 1^36,37^. Our PBMC median (IQR) cellular AR for NMR was 0.998 (0.48–27.05) but the value demonstrated high variability among rats. Overall, it appears that NMR, in the presence of RTV, shows similar intracellular uptake to other PIs. This finding is important as previous studies have shown that SARs-CoV-2 can infect monocytes and T-lymphocytes^5,38^.

To evaluate specific drug uptake by relevant cells of the CNS rather than only CSF, we investigated the uptake of NMR and RTV in astrocytes, pericytes and neurons individually (Fig. 1). We found that the uptake of NMR and or RTV in the presence of the other drug differed significantly in neurons, astrocytes and pericytes. For neurons, the presence of RTV increased the uptake of NMR significantly. This increased uptake effect on NMR is likely a result of efflux transporter inhibition (p-glycoprotein [P-gp]) by RTV^39^. A study by Eng and colleagues looked at efflux transporter inhibition effects on NMR using Caco-2 cell monolayers^40^. They showed that inhibition of Breast Cancer Receptor Protein (BCRP) and Multi-Drug Resistance 1 (MDR1) enhanced the apparent permeability of NMR from 0.80 ± 0.15 to 4.05 ± 0.26 cm/s in Caco-2 cell monolayers^40^. Specific to CNS, a study by Ghosh and colleagues looked at cellular localization and functional significance of cytochrome P450 3A4 (CPY3A4) and MDR1 in the CNS and found coexpression by BBB endothelial cells and neurons showing potential implications on drug metabolism and cytoprotective mechanisms^41^. As RTV is a substrate to many of the efflux transporters (relevant to the BBB), we predict similar effects of RTV on NMR permeability through the BBB^42^. The expression of P-gp in human brain capillary endothelial cells is well documented^43^. However, its expression in astrocyte, pericytes, and neurons is still under investigation^44–47^. No significant differences were noted in astrocytes and pericytes when NMR was administered alone or with RTV (Fig. 1a). More investigation is needed to further substantiate our hypothesis of the RTV-P-gp-NMR transporter interaction. Further studies by our group will evaluate NMR/RTV penetration utilizing a more novel 4-cell in vitro model, with transporter expression^48^. When comparing NMR to other PIs used to treat HIV, as a class, they achieve poor CSF exposure^49^. However, when co-administered with RTV (or other boosters), CSF penetration has been shown to increase^50,51^. For example, increased CSF concentrations of atazanavir were found when administered with RTV (7.9 to 10.3 ng/mL)^52^. When looking at RTV in our in vitro model, uptake by astrocytes was high at 41.97% and moderate by pericytes at 20.2%. In the presence of NMR, RTV uptake increased to 66.65% in astrocytes and 32.19% in pericytes. RTV CSF distribution is low^53–55^. To our knowledge, there are no prior studies for human neuronal uptake of RTV, and our results indicated moderate neuronal entry of RTV in the presence or absence of NMR (Fig. 1b). Nevertheless, a CNS drug delivery experiment for RTV in a mouse model showed moderate penetration of RTV in brain parenchyma tissue^56^. Additional characterization of RTV uptake for human neuronal tissue is desired. A study by Anthonypillai and colleagues in guinea pigs found that CSF levels of RTV were low, but RTV levels in the choroid plexus and brain were higher^53^. They hypothesized this was due to RTV regulation in the CSF and choroid plexus by efflux transporters that may limit drug accumulation in the CSF. In our study, we found that RTV uptake in astrocytes and pericytes was affected by NMR (Fig. 1b). Thus, we believe this is likely due to NMR’s effect on associated transporters. Transporter inhibition studies are warranted to provide insight on the mechanisms behind the differences seen between cell lines. It is relevant to note the BCSFB is considered “leakier” compared to the BBB and transport across is inversely related to molecular weight of the compound. This is due to the BCSFB being comprised of ependymal cells of the choroid plexus. This results in looser tight junctions compared to those found in the BBB. As such, drug penetration into the CSF is not an index of BBB transport, but rather a measure of transport across the choroid plexus at the BCSFB.

We developed a 3-compartment PK model to predict individual animal concentration–time profiles for plasma and CSF, as shown in Fig. 2. This allowed us to accurately predict CSF and plasma exposures, which were used to calculate CSF penetration (Table 2). This also allowed us to make comparisons of our PK estimates with clinical and animal data. For example, the median half-life for NMR in the presence of RTV for the rats was 2.4 × faster than what is seen in humans (2.55 h vs. 6.05 h)^9^. This is expected as smaller animals clear most drugs faster given the principles of allometry^40,57^. When comparing our NMR half-life to other animal models for NMR, we found that our half-life estimation was within the range of other oral rat PK studies (10 mg/kg: 4 h [range: 2.9–5.1], 10 mg/kg: 2.8 h ± 1.4 h)^40,58^. Our estimation for time at which C_max_ is first observed (T_max_), was similar to other rodent models (mean: 1.84 h vs. mean: 1.5 h)^40,58^. The median relative F value of 54.5% in our study was also consistent with other literature values estimated in rats for NMR (34–50%)^40^. We note our animals were not restricted of food or water, and this is likely why we saw variability in F between and within animals (Table 1b: range: 32–62%). For NMR Ka, our model estimate of 0.51 h^−1^ was also in agreement with finding reported by others (0.55 h^−1^)^34^. We compared our values for C_max_ and AUC with clinical data from healthy volunteers. Rat geometric mean plasma values for C_max_ (2.48 µg/mL or 2480 ng/mL) and AUC_daily average_ (20.25 µg*hr/mL) compared well with healthy human geometric mean values of C_max_ (2.21 µg/mL) and AUC_0-12 h_ (23.01 µg*h/mL) supporting our allometric dose scaling strategies^34^. Our AUC estimation was a daily average given the difficulty of standardizing twice-daily dosing in animals and the healthy volunteer data was based on an AUC of 0–12 h. When comparing our rat CSF concentrations to the PBPK modeling performed by Saleh and colleagues, our CSF C_max_ (median 0.41 mg/L or 410 ng/mL) is in agreement with what was projected in human brain extracellular fluid (~ 0.3–0.44 mg/L, points extrapolated using graphgrabber 2.02)^32,59^. This shows the potential clinical application of our rat model as we were able to humanize C_max_ exposure in both plasma and CSF. Last, it is important to also mention NMR’s lipophilicity in relation to BBB penetration. A drug metabolism study on NMR disposition indicated that it is moderately lipophilic with a lipophilicity coefficient (LogP) of 1.68, showing low passive apparent permeability (P_app_) of 1.76 × 10^−6^ cm/s^40^. Utilizing different cell lines (i.e., Caco-2 cell monolayers), NMR exhibited similar trends of low permeability across the monolayer barrier^40^. Our findings of low penetration of NMR through the BBB are also in agreement with Lipinski’s rule of five that postulates a lipophilicity range of 2.0 to 3.5 is a fundamental predictor for BBB penetration via passive diffusion^60^. Other PIs exhibit a range of lipophilicity from 1.0 to 5.69 depending on specific physiochemical properties^61,62^.

Our study has limitations. First, we did not design this study for animals infected with SARS-CoV-2 and thus could not assess viral loads in the CSF vs. CSF concentrations of NMR. Because our findings indicate that CNS levels of NMR may not be adequate to achieve therapeutic concentrations, plans for utilizing an infection model with the golden Syrian hamster model are ongoing. Second, our tissue concentrations represent total drug concentrations based on homogenized tissues. Understanding the dynamic relationship of unbound tissue concentrations vs. time or site-specific tissue concentrations would require microdialysis or other techniques. Further, it is unknown if CSF catheter placement could have influenced CSF penetration or if concentration-mediated changes to CSF transit occur. Future work to address concentration- mediated penetration utilizing a BCSFB in vitro model is planned^48^. In addition, it is unclear how our animal model compares to active infection where inflammation could increase drug penetration through the BBB in active SARs-CoV-2 infection. In this context, a recent review on BBB integrity alteration by SARs-CoV-2 pointed to the increased expression of matrix metalloproteinase-9 (MMP9) in COVID-19 infection. The increased MMP9 activates RhoA (Ras homolog family member A), causing more degradation of type IV collagen of the BBB basement membrane and altering the barrier's integrity^63^. Moreover, in our in vitro experiment for cellular uptake of NMR/RTV by human brain cells, we did not include human brain microvascular endothelial cells (hBMECs) because our earlier finding suggested no infection of hBMECs by SARs-CoV-2, while we observed high infection in human astrocytes and pericytes^18^. This was consistent with the lack of ACE-2 receptor expression in hBMECs, when compared to astrocytes and astrocytes. Additionally, the cells were not available at the time of these experiments. Differences in the expression of BBB transporters (i.e., P-gp) among species exist, and variations could result in differences in clinical extrapolation. A study by Morris and colleagues showed cross species expression of BBB transporters and that rats and humans have many of the same transporters present^64^. However, describing species differences in transporter expression is a difficult task given all the potential transporters involved with NMR and RTV and was beyond the scope of this study. A mechanistic study by Verscheijden and colleagues used PBPK modelling and calculated plasma concentration-corrected brain concentrations (Kp) values for humans and rodents specific only for P-gp correction for various medications^65^. We acknowledge the complexity of the BBB transporter expression between species. However, regardless of transporter differences, our dosing achieved humanized exposures in plasma and CSF. Further studies specific to transporter expression are warranted and planned. Also, we quantified total NMR concentrations and did not quantify free drug (NMR is 69% protein bound)^9^. CSF penetration via plasma to CSF estimation should also consider free drug in the plasma as drug found in the CSF is unbound to proteins, and future studies might quantify free NMR concentrations to capture this consideration more accurately. The PD endpoints we utilized for CSF, plasma, tissues, and PBMC were adjusted and unadjusted for plasma protein-binding, depending on the matrix. Last, our final model estimation of K_30_ denotes overall elimination of NMR from the CSF, including uptake by various types of cells in the CNS. This may be an oversimplification and a more mechanistic description should be evaluated utilizing an advanced quantitative systems pharmacology approach.

In this study, we determined NMR CSF and CNS penetration utilizing in vitro and in vivo models and quantitatively described the transit of NMR from plasma to the CSF. In addition to NMR, molnupiravir and remdesivir are two other antiviral agents for the treatment of SARS-CoV-2. Similarly, CNS penetration data for them are lacking. The data from our in vivo rat model demonstrates that NMR penetration into CSF and CNS tissues may be inadequate. Our in vitro model data shows minimal NMR uptake into cells relevant to the CNS. Collectively, these findings may have implications for viral persistence in these compartments and neurologic post-acute sequelae of COVID-19. These data motivate future investigations utilizing an infection model to understand the pharmacodynamic effects of NMR drug concentrations in the CNS on viral loads in the CNS. If longer treatment or higher doses correspond to increased NMR penetration in the CNS, decreased viral loads, and decreased CNS inflammation, they provide a basis to investigate alternative dosing strategies. This information would be fundamental for optimizing treatment of Long COVID-19.

## Methods

This study was conducted at the University of Nebraska Medical Center in Omaha, NE. All study methods were approved by the Institutional Animal Care and Use Committee (IACUC; Protocol #2006507) and conducted in an AAALAC-accredited animal facility. This study was reported in accordance with ARRIVE guidelines.

### Chemicals and reagents

Animals were administered NMR/RTV (NMR: Medkoo Biosciences, Catalog#555985 Lot#: C22R06B23, Morrisville, NC, USA. RTV: Medkoo Biosciences, Catalog#318671, Lot#: A22M08B04) for oral dosing. Artificial CSF (TOCRIS Biotechne, #3525) and normal saline (B/BRAUN, Lot#: R5200-01) were used as described in sampling methods below. LC–MS/MS standard curves were generated using commercially obtained NMR (Cayman Chemical, Lot#:0635075, Ann Arbor, MI, USA) with a purity of > 98%. Nirmatrelvir-2H9 (2H9-PF-07321332, Lot#: NA-ALS-22-044-P3, Alsachim, Illkirch, France) was used as an internal standard for the NMR quantification. Formic acid, methanol and acetonitrile were obtained from Fischer Scientific (Waltham, MA, USA). Ultra-pure water was obtained from UNMC via a Barnstead GenPure xCAD Plus water purification system (Thermo-Fisher, Waltham, MA, USA). Frozen, non-medicated, non-immunized, pooled Sprague–Dawley rat plasma and pooled human CSF (BioIVT, Westbury, NY, USA) were used for calibration of standard curves. For oral dosing, NMR and RTV were mixed into a premade vehicle formulation similar to previous methods^40,58,66^.

### Cells and culture system

Human brain primary astrocytes (#1800), pericytes (#1200), and human neurons (#1520) were purchased from ScienCell Research Laboratories (SCRL), USA. Required media and growth supplements for the respective cells were also obtained from SCRL. Astrocytes were cultured in astrocyte media (AM) (Catalog#1801) and astrocyte growth supplement (AGS) (Catalog#1852); pericytes were cultured in pericyte media (PM) (Catalogue#1201), pericyte growth supplement (PGS) (Catalog#1252) and human neurons were seeded in neuronal media (NM) (Catalog#1521) with neuronal growth supplement (NSG) (Catalog#1562). Supplements, including FBS (Catalog#0010), and penicillin/streptomycin solution (P/S) (Catalog#0503) were also purchased from ScienCell. Frozen cells were revived and cultured according to the manufacturer's instructions. Astrocytes and pericyte cells were grown in either a 25 cm^2^, 75 cm^2^, or 150 cm^2^ culture flask (TPP#90076) in accordance with experimental requirements. Culturing flasks were pre-coated for human brain cells with bovine fibronectin at 2 µg/ml (ScienCell#8248). The 6-well plates (TPP#92006) were coated with Poly-L-Lysine (Sigma#RNBL4935) for 10 min at room temperature for human neuron cells, washed with PBS, and air dried. Astrocytes and pericytes were harvested by trypsinization (0.25% trypsin, Lonza#CC-5012) from the flasks having close to 90% confluency of growing cells and washed in DPBS (Dulbecco’s#1960454). Cells were prepared for counting by mixing 10 µl of cell suspension with 10 µl of trypan blue. 10 µl of the mixture was read in a cell counter (Invitrogen Countess). Neuron cells were directly seeded on the pre-coated 6-well plates after thawing the frozen vials.

### Cell seeding in 6-well plates

All steps were carried out in a biosafety cabinet under aseptic conditions, similar to methods previously described^48^. Astrocytes with a cell count of 0.5 × 10^6^/well were seeded into 6-well plates containing 2 ml of astrocyte media in each well. Seeding was performed in triplicate for each drug or drug combination and incubated in 37 °C cell culture incubator, as described in our previous work^48^. Pericytes with a cell count of 0.5 × 10^6^/well were seeded into 6-well plates containing 2 ml of pericyte media in each well. Cell seeding was performed in triplicate for each drug or drug combination and incubated in 37 °C cell culture incubator. Neurons with a cell count of 0.3 × 10^6^/well were seeded into 6-well plates containing 2 ml of neuronal media in each well. Seeding was performed in triplicates for each drug or drug combination and incubated in 37 °C cell culture incubator.

### Drug formulation for in-vitro work

Powdered NMR (Medkoo Biosciences, Catalog#555985, Lot#: C22R06B23) was dissolved in 1 mL of 100% DMSO to make a stock concentration of 4.4 mg/mL. Powdered RTV (Medkoo Biosciences, Catalog#318671, Lot#: A22M08B04) was dissolved in 1 mL of 100% DMSO to achieve the stock concentration of 2 mg/mL. NMR and RTV were weighed and dissolved in 1 mL of 100% DMSO to achieve 4.4 and 2 mg/mL stock concentration for NMR and RTV, respectively.

### Drug addition to cells and sample preparation

NMR and RTV, individually or in combination, were added to the cultured cells at 2200 ng/mL and 1000 ng/mL final concentration, respectively. The in vitro doses of NMR and RTV were selected based on previous studies^67,68^. After 24-h incubation with drugs, astrocytes, pericytes, and neuron cells were washed once with PBS and harvested using a cell scraper (Corning #3010) in 500 µl of 70% methanol. Samples were kept at − 20 °C prior to drug quantification.

### Experimental design and animals

Male Sprague-Dawley rats (n=10, mean weight=306 g, age=~65–70 days) were obtained from Charles River (Raleigh, NC 27610). All catheters (cisternal and vein cannulation) for the animals were surgically implanted^69,70^ at Charles River prior to shipping. On arrival to the housing facility, animals were acclimated for 72 hrs prior to starting study protocol. Catheter management was performed daily to ensure viable sampling. Animals were administered 30 mg/kg NMR + 10 mg/kg RTV twice a day (60 mg/kg NMR and 20 mg/kg RTV total daily dose) daily for five days (as described below). All NMR/RTV doses were administered orally via gavage. The dose chosen for this study was allometrically scaled to a humanized equivalent of NMR/RTV based on fixed dosing (i.e., 60 kg patient, 300 mg NMR + 100 mg RTV twice daily, = 10 mg/kg NMR + 3.33 mg/kg RTV daily x scaling factor of 6.2 = ~60 mg/kg NMR + ~20 mg/kg RTV daily)^57^. The five day duration of the study also aligns with the current FDA recommendation for treatment of COVID-19 with NMR/RTV in patients^10^. Rats were housed in a light and temperature-controlled room for the duration of the study and allowed free access to water and food, except during sampling. Data were analyzed for all animals that entered the protocol. When animals contributed incomplete data (i.e., early protocol termination), all available samples were analyzed for PK. Concentrations below the lower limit of quantification were inputted as 0^71^.

### Blood, CSF, PBMC, and tissue sampling and determination of NMR concentrations

Blood samples were drawn from a single right-sided internal jugular vein catheter in a sedation-free manner when possible. Blood catheter lines were flushed with normal saline after each blood draw to prevent blood contamination. CSF was collected via an intracisternal catheter. Isoflurane gas was used for temporary sedation when needed (5% initially, followed by 1–3% maintenance). Within each 24 hrs, planned sample collection was eight blood and two CSF samples per animal. An approximation of the full sampling strategy over the five day study can be found in Supplemental Fig. 6. Each sample obtained (0.25 mL blood and 0.05-0.1 mL CSF aliquots) was replaced with either an equivalent volume of normal saline or artificial CSF (as appropriate) to maintain euvolemia. Blood and CSF samples from NMR-treated animals were processed similar to our previous reports^72–75^.

Upon completion of the protocol, rats were euthanized, and tissues (lungs, heart, kidney, brain, liver) were harvested. The tissues were perfused, rinsed with cold saline solution, blotted with paper towel, and snap-frozen. Rat tissues (lungs, heart, kidney, brain, liver) were analyzed for NMR content by preparing tissue homogenate samples. PBMC sampling was conducted on each rat prior to termination using mononuclear cell preparation tubes per manufacture protocol (BD Biosciences, Franklin Lakes, NJ).

Plasma, CSF, tissue, and PBMC concentrations of NMR were quantified with LC–MS/MS using individual standard curves for each matrix (ranges: CSF, 1–250 ng/mL; plasma, 20–10,0000 ng/mL; PBMC, 0.01–5 ng/mL). Standard calibrators, quality controls, and samples were prepared in microcentrifuge tubes. Internal standard was added to track the analyte of interest through the extraction and instrumentation processes. NMR was extracted from 20 μL of rat plasma, PBMCs or CSF with a stable labeled internal standard [2H9]-PF-07321332 (IS) by a protein precipitation using 50:50 ACN:MeOH to provide a protein free extract. CSF samples were treated with ammoniated methanol prior to extraction to ensure no analyte adsorbs to the tube wall as previously described^74^. Supernatant was removed and diluted with mobile phase in a 96-well plate prior to injection. HPLC was used to separate the analytes from potential interferences on a C18 100 × 3.00 mm column (MAC MOD, Chadds Ford, PA, USA) for stationary phase, using 60% acetonitrile, 0.1% formic acid in water as an isocratic mobile phase. Detection of NMR and the IS in plasma and CSF was done with an ABSciex 5500 Q-trap mass spectrometer (ABSciex, Framingham, MA, USA) in positive ion mode. PBMC levels were converted to µM concentrations based on the single cell volume for PMBCs^76^. The assays were linear between plasma concentrations of 20 and 10,000 ng/mL, CSF concentrations of 1 and 250 ng/mL, and PBMC concentrations of 0.01 and 5 ng/mL. The plasma component underwent a complete validation and had a precision of < 4.73 for all measurements, including intra- and inter-assay measurements. Briefly, all bioanalyses were within the pre-determined acceptance criteria of +/− 15% for each level (+/− 20% for LLOQ)^77^.

Tissues were homogenized using a Precellys Evolution Cryolys homogenizer (Bertin Technologies, Montigny-le-Bretonneux, France). Each tissue was homogenized with 0.5 mL of 70:30 methanol: 25 mM phosphate buffer. Calibration curves for the tissue homogenates were prepared as described above in the section on estimation of NMR in plasma. Tissues were quantified by weight (mg of drug/g of tissues), reported as mg/g, and converted to mg/mL as previously described^72,78^. Calibration curves for the tissue homogenates were prepared as described above. All units were reported in ng.

PBMC cellular and tissue AR were calculated as observed NMR PBMC and tissue concentrations to NMR plasma concentrations at the same time of collection^36,37^.

### NMR PK and drug exposure

The simplest base PK model considered was a 3-compartment model with an oral compartment (first order absorption), plasma compartment, and a CSF compartment. Three and four-compartment models with/without a lag constant were similarly fit using the nonparametric adaptive grid (NPAG) algorithm within the Pmetrics package version 1.5.0 (Los Angeles, CA) for R version 3.2.1 (R Foundation for Statistical Computing, Vienna, Austria)^79,80^. Multiple different CSF models were considered where CSF intercompartmental clearance (CL)/transfer and CSF CL were added and omitted based on investigator judgement, and other PK CSF studies^81–84^. A model comparison table can be found in Supplemental Table 1. The initial estimate of parameter weighting was accomplished using the inverse of the assay variance. Model performance was quantitatively described using observed vs. predicted concentrations to calculate bias, imprecision, and coefficients of determination^85^. The final model was selected based on regression of observed vs. predicted concentrations, prediction bias, visual plots of parameter estimates, lowest -2LL/Akaike information criterion and rule of parsimony. We modeled the relative bioavailability (F) for each dose in a given rat to account for inter-occasion variability in concentrations among doses, by taking the maximum post-dose peak concentration observed for that rat over all doses and calculating F for each dose as the peak after that dose divided by the maximum peak. The dose which was followed by the maximum peak then had F=1, and all other doses were F≤1.

To compare NMR concentrations in animals to a putative PD endpoint, the concentration needed for three times the 90% maximal effective concentration (3xEC_90_) for the SARS-CoV-2 was utilized^33^. The FDA integrated review from the clinical studies (EPIC-HR) showed 95% of participants had NMR trough concentrations ≥3xEC_90_^34^. Therefore, the goal for the CSF was set to achieve the same exposure conditions as for plasma. The plasma EC_90Adjusted_ concentration for plasma is 292 ng/mL (585 nM), and the EC_90Un_adjusted_ for CSF is 90.5 ng/mL. Therefore, the 3xEC_90_ PD values would be 876 ng/mL for plasma and tissue, and 271.5 ng/mL for CSF. This EC_90_ value is based on the study on bronchial epithelial cells infected with USA_WAI/2020 isolate^58^.

### Estimation of PK exposure and percent (%) CSF penetration

The best-fit model was used to calculate median maximum a posteriori probability Bayesian NMR plasma and CSF concentration estimates at 12-min intervals over the 5-day study period using each animal’s measured NMR concentrations, exact dose, and dosing schedule. From these concentrations we calculated the AUC__0-5 days_ over the entire experiment using “makeAUC” function within Pmetrics^79,86^. C_max_0-5 days_ from the 12-min interval Bayesian estimates was determined to be each animal’s C_max_0-5 days_.

Ratios of the estimated AUC_csf_ /AUC_plasma_ and C_max___csf_ /C_max___plasma_ were used to determine percent CSF penetration^81,87–90^. AUC was standardized to AUC_0-24 h_ by dividing AUC__0-5 days_ by 5 (i.e., 5-days protocol) to provide an estimated AUC_0-24 h_ value. For C_max___0-5 days,_ the highest predicted CSF concentration and corresponding plasma concentration were used to calculate precent penetration. Only animals with CSF concentrations sampled were used for estimation of CSF penetration.

### Statistical methods

Summary statistics were calculated using GraphPad Prism V7.02 (GraphPad Software Inc., La Jolla, CA). Nonparametric summary statistics were reported given the small sample size and distribution of data.

### Supplementary Information

Supplementary Information.

## Data Availability

The raw data for the drug concentrations are available in the Supporting Data Values file. Raw data for the manuscript are also available from the corresponding author upon request.
