# Supplementary material for: In-vitro and in-vivo assessment of nirmatrelvir penetration into CSF, central nervous system cells, tissues, and peripheral blood mononuclear cells
Source: Sci Rep. 2024 May 10;14:10709. doi: 10.1038/s41598-024-60935-5 (PMC11087525; doi:10.1038/s41598-024-60935-5)
Supplement: Supplementary file 1 — Supplementary Information. [file 41598_2024_60935_MOESM1_ESM.docx]

**Supplemental Figure 1.**

**X_3_**

**V_CSF_**

**CSF Compartment**

**X_2_**

**V_C_**

**Central Compartment**

**K_el_**

**K_23_**

**Ka**

**K_30_**

Differential Equations

*dx_1_(t)/dt* = -Ka*X_1_

*dx_2_(t)/dt* = Ka*X_1_ – (K_el_ + K_23_)X_2_

*dx_3_(t)/dt* = K_23_*X_2_ - K_30_*X_3_

**X_1_**

**Oral Compartment**

Legend: Schematic and differential equations of base three-compartmental PK model. There was an oral compartment (X_1_), central compartment (X_2_), and CSF compartment (X_3_). Drug transit into and out the CSF was depicted with a one-way rate constant (K_23_) and a CSF elimination rate constant (K_30_).

Note: K_30_ represents overall elimination from the CSF, including uptake by various types of cells in the CNS.

Abbreviations: PK=pharmacokinetic, CSF= cerebrospinal fluid, Kel=elimination rate constant, V_c_=volume central compartment, V_csf_=volume cerebral spinal fluid compartment, Ka=rate constant for absorption, K_23_=rate constant to cerebral spinal fluid from central compartment, K_30_=elimination rate constant from CSF compartment, X_1_= amount in the oral compartment, X_2_=amount in the central compartment, X_3_= amount in the CSF compartment

**Supplemental Figure 2**.

(a). (b).


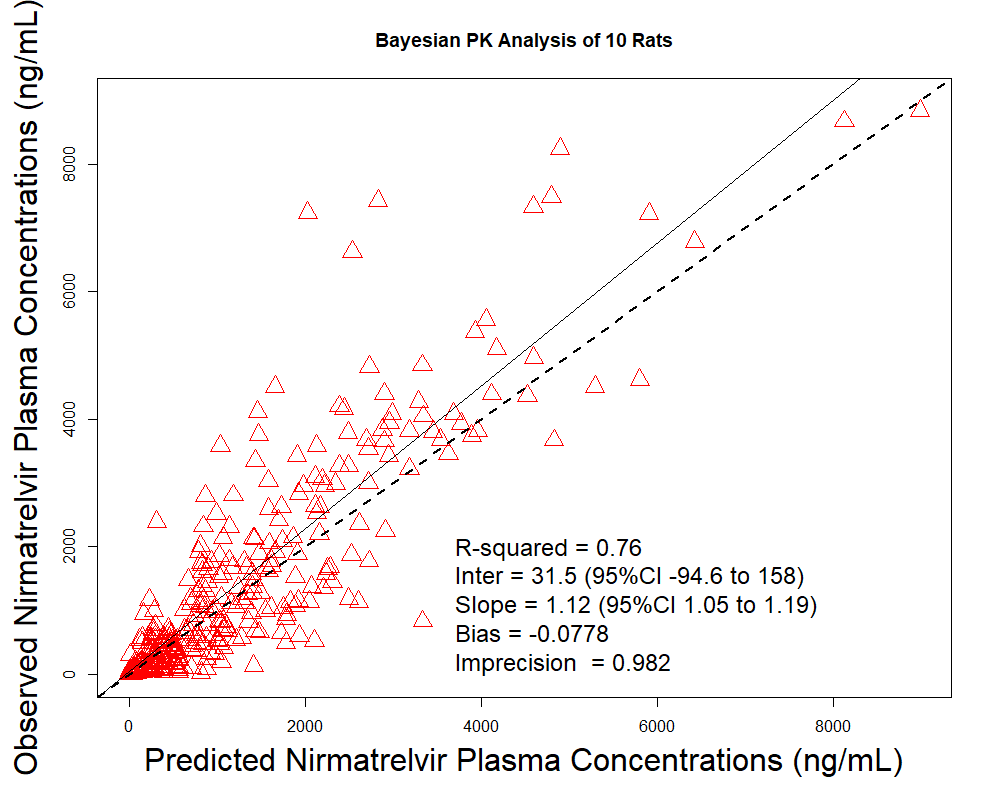

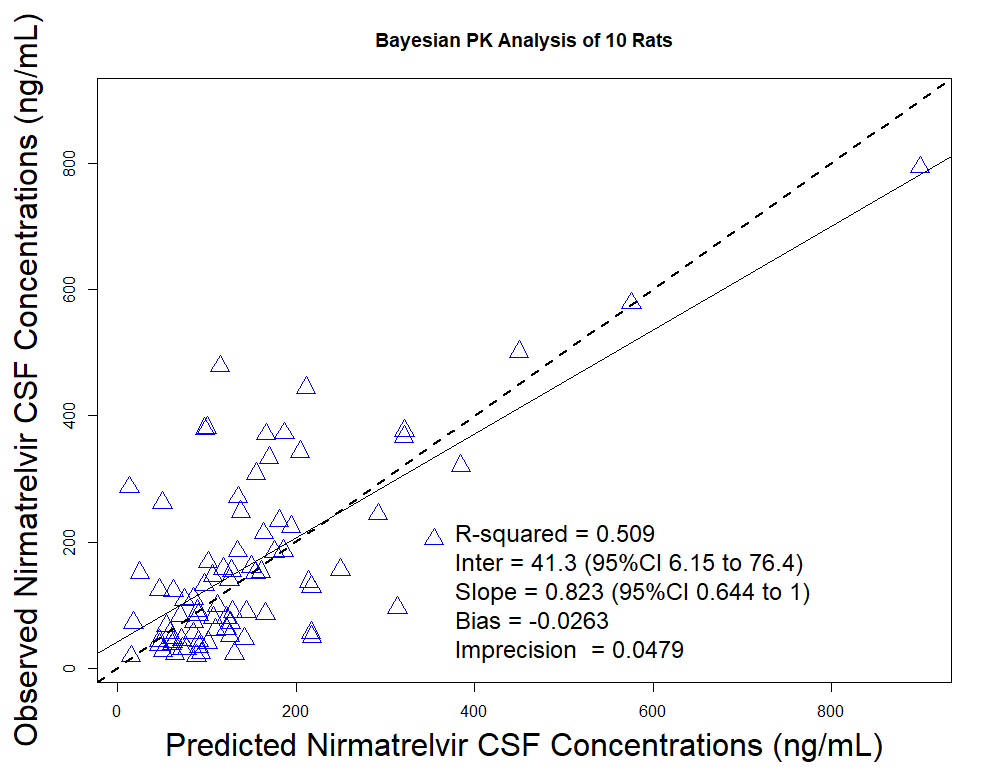


Legend: Observed vs. predicted Bayesian plots from the final model for plasma (a) and CSF (b). Overall, the model fits showed minimal bias and imprecision for both plasma and CSF. The observation variance was proportional with an additive (lambda) model (error = SD^2^ + lamda^2^)^0.5^ where SD for each matrix= C_0_ + C_1_Y (with inputs of C_0_ = 0.1 mg/L and C_1_ = 0.15 in plasma and inputs of C_0_ = 0.01 mg/L, C_1_ = 0.15 in CSF where; Y was = the observed concentration).

*Observed and predicted drug concentrations on above plots converted to ng/mL for consistency (plot diagnostics, [i.e., inter, slope, bias, imprecision] and error model kept in mg/L).

Abbreviations: PK=pharmacokinetic, CSF=cerebrospinal fluid

**Supplemental Figure 3.**


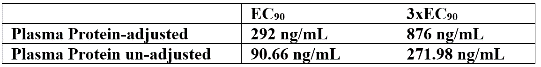


| **Rat** | **Liver (ng/g)** | **Brain (ng/g)** | **Lung (ng/g)** | **Kidney (ng/g)** | **Heart (ng/g)** | **PBMC** (ng/mL)** |
| --- | --- | --- | --- | --- | --- | --- |
| 1 | 11.53 | - | - | 48.1 | - | 14.99 |
| 2 | 107.9 | 51.37 | - | 466.4 | - | 674.33 |
| 3 | 106 | 4.17 | 80.67 | 28.82 | 53.94 | 31.97 |
| 4 | 38.56 | - | 18.71 | 94.47 | 10.07 | 189.81 |
| 5 | 26.91 | 32.02 | 13.56 | 16.88 | 15.01 | 500.5 |
| 6 | 108.72 | 10.95 | 55.05 | 126.33 | 89.27 | 31.47 |
| 7* | 3850.04 | 98.38 | 1442.59 | 822.59 | 647.73 | 0 |
| 8 | 133.8 | 33.27 | 43.91 | 51.63 | 69.5 | 61.44 |
| 9 | 285.53 | 15.63 | 124.96 | 240.89 | 118.95 | 110.39 |
| 10 | 820.4 | 10.93 | 69.53 | 101.21 | 52.73 | 56.94 |
| Median (IQR) | 110  (35.65-419.5) | 23.83  (10.94-46.85) | 67.86  (25.01-111.3) | 97.84  (43.28-297.3) | 61.72  (24.44-111.5) | 61.44  (31.7-345.2) |

Legend: Violin plots of tissue and PBMC Concentrations of NMR. Compared to all the tissues, the brain had the lowest median NMR concentrations which were all <3xEC_90_ regardless of adjustment for plasma protein binding. Figure depicts EC_90adjusted_ (black dotted line) values for reference. Tissues quantified in grams, mg/g of drug in tissue is equivalent to a concentration of mg/mL. Values left unrounded given the low concentrations. Units reported in ng/mL.

*Rat 7 only completed 1 day of treatment, no PMBC levels available, rats 1 and 2 did not have lung or heart NMR concentrations due to tissue processing complications.

**PBMC levels converted to ng/mL

Abbreviations: BLOQ=below level of quantification, IQR=interquartile range, EC_90_= 90% maximal effective concentration.

**Supplemental Figure 4.**

(a)


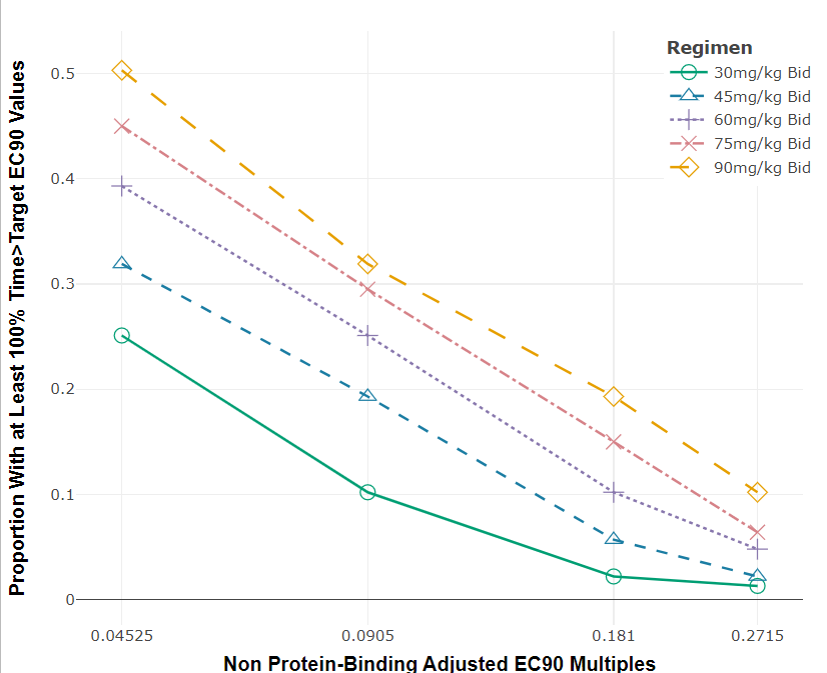


(b)


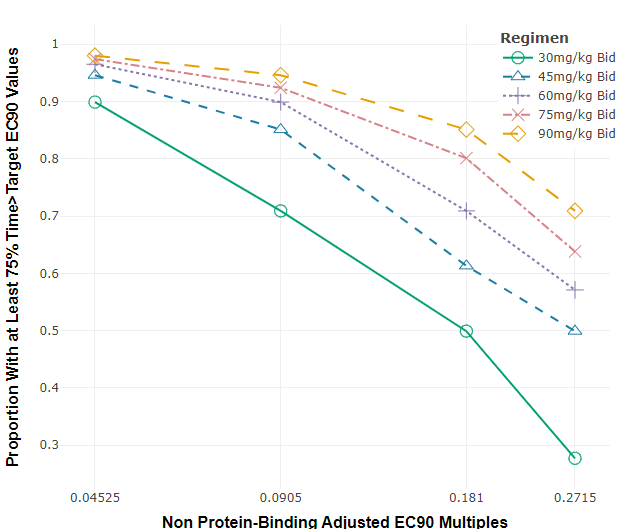


(c)


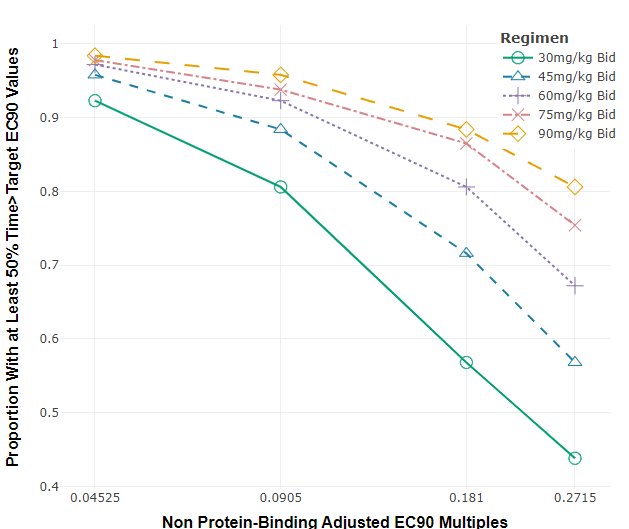


Legend: Monte Carlo simulations (n=1000, assuming a 300g rat, fu=1) showing different proportions of target attainment in CSF at 100% (a), 75% (b) and 50% (c) of time>EC_90Un_adjusted_ multiples at various doses of NMR (mg/kg/BID). Doses>90mg/kg BID would be needed in rats to achieve 100% of time>3X EC_90Un_adjusted_ in CSF. X axis represents non protein-binding adjusted EC_90_ multiples (where 0.0905 = EC_90_ [units in mg/L]).

Abbreviations: CSF=cerebrospinal fluid, EC_90_= 90% maximal effective concentration, fu= fraction unbound

**Supplemental Figure 5.**

(a)


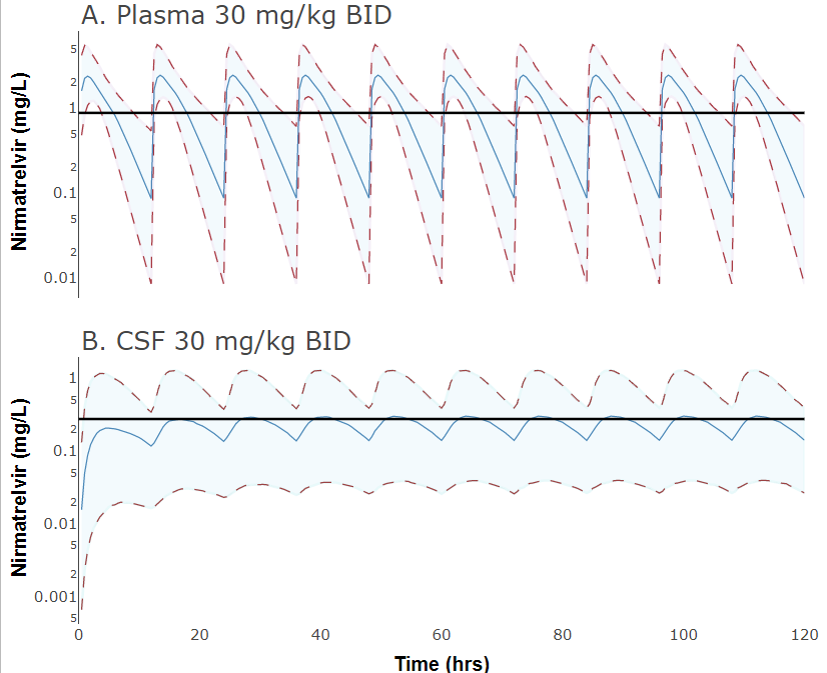


**0.2715 mg/L**

**0.876 mg/L**

Log-transformed


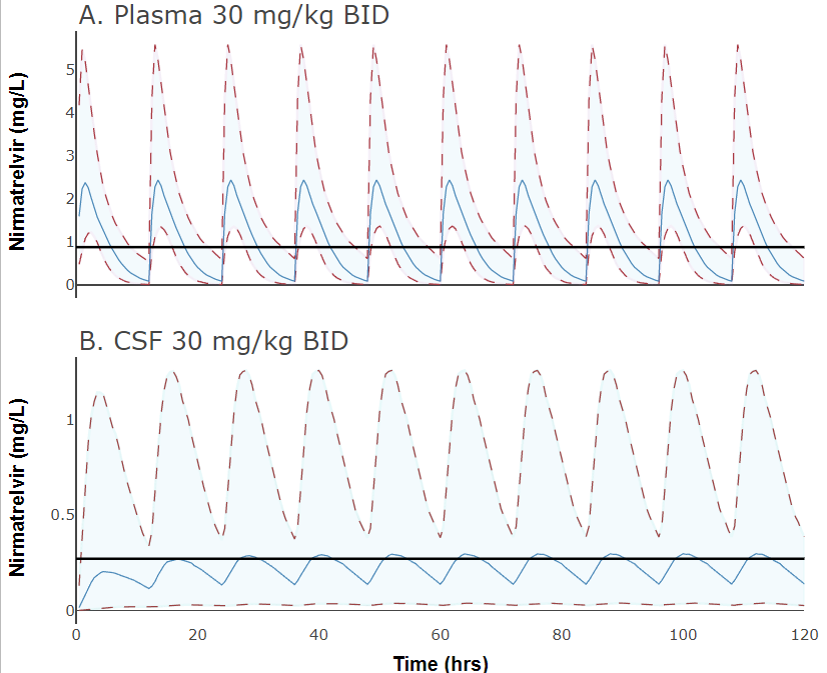


**0.2715 mg/L**

**0.876 mg/L**

Linear

(b)


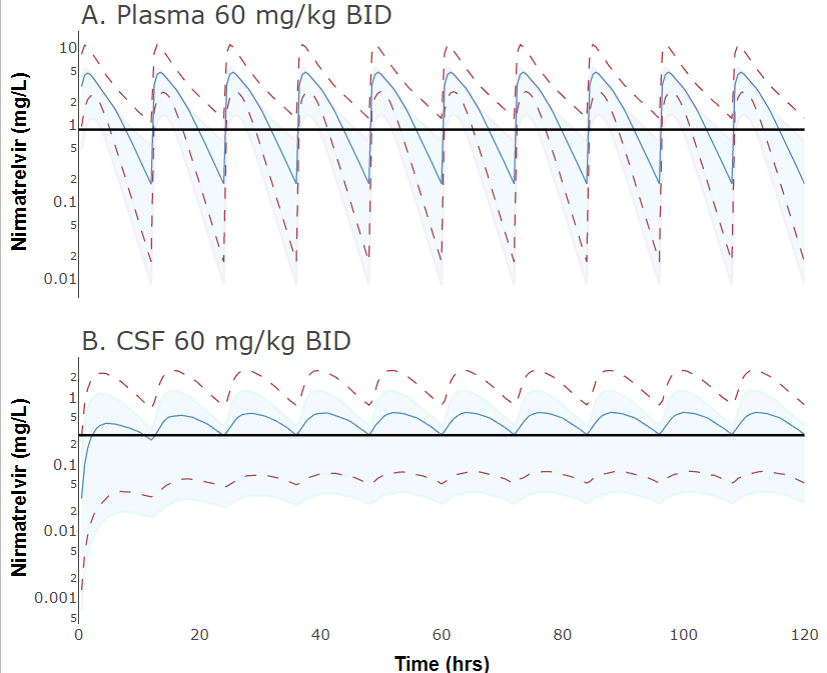


**0.2715 mg/L**

**0.876 mg/L**

Log-transformed


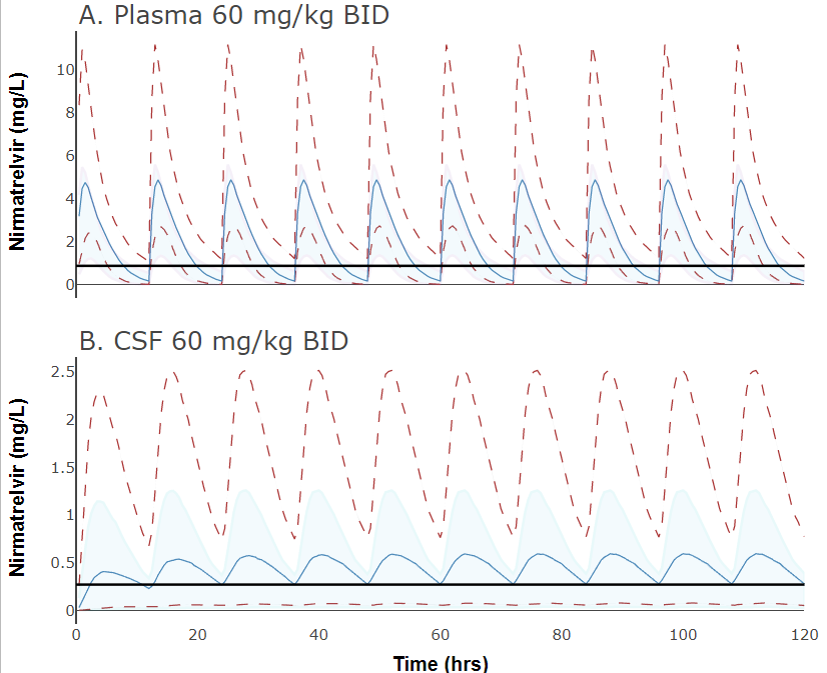


**0.2715 mg/L**

**0.876 mg/L**

Linear

(c)


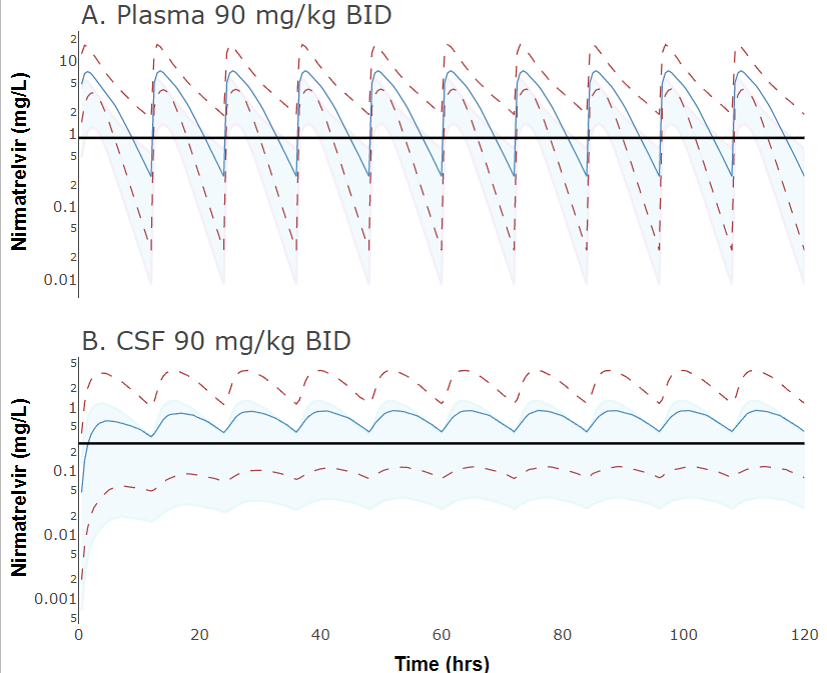


**0.2715 mg/L**

**0.876 mg/L**

Log-transformed


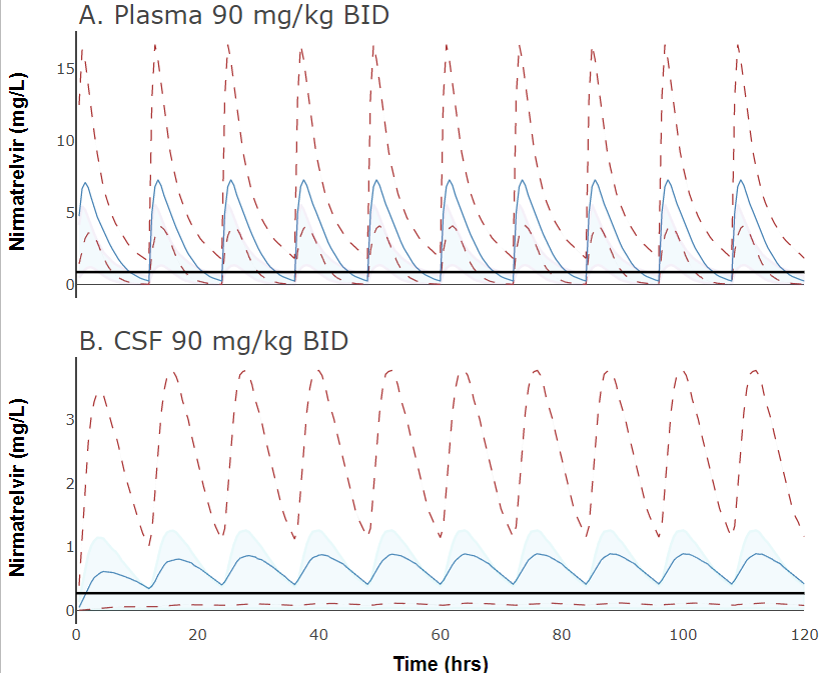


**0.2715 mg/L**

**0.876 mg/L**

Linear

Legend: Plasma (a) and CSF (b) linear and log-transformed predictions of NMR at 3 different doses (30, 60 and 90 mg/kg BID) based on the Monte Carlo simulations (n=1000, assuming 300g rat, fu=1). Median (solid blue line) and 95 percentiles (red dotted lines with blue shaded area) of plasma and CSF NMR PK profiles are depicted. The PK profiles are depicted against the 3xEC_90_ (black line) for both plasma protein adjusted (0.876mg/L) in plasma and unadjusted (0.2715mg/L) in CSF. Units kept in mg/L.

Abbreviations: NMR=nirmatrelvir, EC_90_=90% maximal effective concentration, BID=twice daily, CSF=cerebrospinal fluid, fu=fraction unbound

**Supplemental Figure 6**.

|  |  | |  | | | |  | | | | | | | | | | | | | | | | | | | | | | | | | | | |  |
| --- | --- | --- | --- | --- | --- | --- | --- | --- | --- | --- | --- | --- | --- | --- | --- | --- | --- | --- | --- | --- | --- | --- | --- | --- | --- | --- | --- | --- | --- | --- | --- | --- | --- | --- | --- |
|  |  | | **Day1** | | | | | | | | | | | | **Day2** | | | | | | | | | | **Day3** | | | | | | | | | |  |
|  | **Blood** | | | | | | | | | | | **CSF**  **CSF** | | | **Blood** | | | | | | | | **CSF** | | **Blood** | | | | | | | | | **CSF** | |
| **Rat** | H (relative to dose) | | | | | | | | | | | H | | H | H (relative to dose) | | | | | | | | H | H | H (relative to dose) | | | | | | | | | H | H |
| **1** | 1 | 4.5 | | 5.5 | 6 | 21.5 | | NA | NA | NA | NA | 5 | | 21.5 | 0.5 | 1 | 2 | 7 | 21 | NA | NA | NA | 7 | NA | 0.5 | 1 | | 2 | 4 | | 5 | 6 | 8 | NA | NA |
| **2**  **3** | 1 | 2.5 | | 4 | 8 | 22 | | NA | NA | NA | NA | 8 | | NA | 1 | 4 | 8 | 21 | NA | NA | NA | NA | 8 | *23 | 1 | 4 | | 7 | 22 | | NA | NA | NA | 1 | 7 |
| **3** | 0.5 | 1 | | 3 | 5 | 7 | | 7.5 | 8 | 9 | 11 | 7 | | NA | 1 | 2 | 5 | 6 | 7.5 | 8 | 9.5 | 13 | 0.5 | 7 | 1 | 2 | | 5.5 | 7 | | 7.5 | 9 | 11 | 8 | NA |
| **4** | 0.5 | 2 | | 4 | 6 | 8 | | 9 | 11 | 13 | 15 | 8 | | NA | 26 | 1 | 2 | 6 | 8 | 10 | 12 | 15 | 26 | 8 | 27 | 0.5 | | 2.5 | 6 | | 8 | 10 | 12 | 27 | 8 |
| **5** | 0.5 | 1 | | 3 | 5 | 5.5 | | 6 | 8 | 9 | 11 | 6 | | NA | 33 | 0.5 | 2 | 5 | 7 | 9 | 11 | 13 | *33 | 8 | 35 | 0.5 | | 1 | 6 | | 8 | 9 | 11 | 35 | 8 |
| **6** | 1 | 2 | | 6 | 9 | 11 | | 13 | 14 | 16 | NA | 2 | | 14 | 1 | 3 | 6 | 10 | 15 | NA | NA | NA | 3 | NA | 1 | 2 | | 6 | 8 | | 10 | 12 | 16 | 2 | 12 |
| **7** | 2 | 4 | | 6 | 8 | 9 | | 12 | 13 | 15 | NA | NA | | NA | NA | NA | NA | NA | NA | NA | NA | NA | NA | NA | NA | NA | | NA | NA | | NA | NA | NA | NA | NA |
| **8** | 5 | 2 | | 5 | 8 | 9 | | 10 | 11 | 13 | NA | 2 | | 11 | 1 | 3 | 6 | 8 | 10 | 11 | 12 | NA | 3 | NA | 1 | 2 | | 5 | 8 | | 10 | 12 | 15 | 2 | 12 |
| **9** | 1 | 2 | | 5 | 8 | 9 | | 10 | 12 | 16 | NA | 2 | | NA | 1 | 2 | 6 | 8 | 11 | 12 | 14 | 18 | 2 | 14 | 1 | 3 | | 6 | 8 | | 10 | 12 | 15 | 3 | NA |
| **10** | 1 | 3 | | 5 | 9 | 10 | | 12 | 13 | 15 | NA | 3 | | 13 | 1 | 3 | 5.5 | 8 | 10 | 14 | 16 | NA | 3 | NA | 1 | 3 | | 5 | 8 | | 10 | 13 | 16 | 3 | 13 |
|  |  | |  | | | | | | | | | | | | | | | | | | | | | | | | | | |  | | | |  |  |
|  |  | | **Day4** | | | | | | | | | | | | | **Day5** | | | | | | | | | | | **Day6** | | |  |  |  |  |  |  |
|  | **Blood** | | | | | | | | | | | | **CSF** | | | **Blood** | | | | | | | | **CSF** | | | **Terminal Draws** | | |  |  |  |  |  |  |
| **Rat** | H (relative to dose) | | | | | | | | | | | | H | | H | H (relative to dose) | | | | | | | | H | H | |  |  |  |  |  |  |  |  |  |
| **1** | 0.5 | 1 | | 2 | 3.5 | 8 | | NA | NA | NA | NA | | 21.5 | | NA | 0.2 | 1 | 2 | 3 | 4 | 6 | 8 | NA | NA | NA | | T | | |  |  |  |  |  |  |
| **2**  **3** | 1.5 | 5 | | 8 | 23 | NA | | NA | NA | NA | NA | | 21.5 | | 32 | 1 | 2 | 6 | NA | NA | NA | NA | NA | *37 | 45 | | T | | |  |  |  |  |  |  |
| **3** | 0.5 | 1 | | 5 | 7 | 8 | | 10 | 13 | 25 | NA | | 27 | | 35 | 0.5 | 1.5 | 4 | 7 | 9 | 11 | 13 | 14 | 9 | NA | | T | | |  |  |  |  |  |  |
| **4** | 25 | 0.5 | | 1 | 4 | 6 | | 8 | 10 | 14 | 15 | | 25 | | 6 | 0.5 | 1.5 | 5.5 | 7 | 9 | 11 | 13 | 14 | 7 | 25 | | T | | |  |  |  |  |  |  |
| **5** | 0.5 | 2 | | 5 | 7 | 8 | | 9 | 14 | NA | NA | | 1 | | 8 | 25 | 0.5 | 1.5 | 5 | 8 | 9 | 10 | 13 | 8 | 25 | | T | | |  |  |  |  |  |  |
| **6** | 1 | 3 | | 5 | 8 | 10 | | 14 | 15 | NA | NA | | 3 | | 10 | 1 | 3 | 6 | 8 | 10 | 12 | 15 | NA | 3 | 10 | | T | | |  |  |  |  |  |  |
| **7** | NA | NA | | NA | NA | NA | | NA | NA | NA | NA | | NA | | NA | NA | NA | NA | NA | NA | NA | NA | NA | NA | NA | | T | | |  |  |  |  |  |  |
| **8** | 1 | 2 | | 4 | 7 | 9 | | 11 | 13 | NA | NA | | 2 | | 9 | 1 | 2 | 4 | 7 | 9 | 11 | 15 | NA | 2 | 9 | | T | | |  |  |  |  |  |  |
| **9** | 1 | 2 | | 3 | 6 | 9 | | 11 | 14 | NA | NA | | 2 | | 10 | 1 | 2 | 6 | 7 | 9 | 10 | 13 | NA | 2 | 9 | | T | | |  |  |  |  |  |  |
| **10** | 1 | 2 | | 3 | 7 | 9 | | 11 | 14 | NA | NA | | NA | | NA | 1 | 4 | 6 | 8 | 11 | 13 | 16 | NA | 4 | 11 | | T | | |  |  |  |  |  |  |

Legend: Example of staggering sampling schematic utilized for study protocol. Each animal received NMR/RTV dose twice daily for 5-days. Time depicted in the figure is relative to previous day 1st dose.

Abbreviations: CSF=cerebrospinal fluid, N/A=not applicable, H=hour, T=terminal, NMR=nirmatrelvir, RTV=ritonavir

**Supplemental Table 1**.PK Model comparison under different conditions and parameters

| **Model** | **-2LL** | **AIC** | **Bayesian**  **Bias**  **mcg/mL**  **(Central)** | **Bayesian**  **Imp**  **mcg/mL^2^**  **(Central)** | **R^2^**  **Bayesian**  **(Central)** | **Bayesian**  **Bias**  **mcg/mL**  **(CSF)** | **Bayesian**  **Imp**  **mcg/mL^2^**  **(CSF)** | **R^2^**  **Bayesian**  **(CSF)** |
| --- | --- | --- | --- | --- | --- | --- | --- | --- |
| **Three-compartment**  **(final model)** | 757.1 | **771.4** | -0.078 | 0.98 | 0.76 | -0.026 | -0.048 | 0.509 |
| **Four-compartment** | 757.2 | 777.7 | -0.082 | 0.99 | 0.76 | NA | NA | 0.497 |
| **Three-compartment w/ Tlag** | 732.8 | 749.2 | -0104 | 0.984 | 0.771 | NA | NA | 0.488 |
| **Three-compartment w/ intercompartment transfer and CSF clearance** | 751.7 | 768.1 | -0.093 | 0.99 | 0.76 | NA | NA | 0.375 |
| **Three-compartment w/ intercompartment transfer, no CSF clearance** | 750.4 | 764.7 | -0.05774 | 0.9962 | 0.76 | NA | NA | 0.49 |

Note: all model comparisons were not significantly different (P-value: >0.1) and thus, final model based on regression of observed vs. predicted concentrations, visual plots of parameter estimates, lowest -2LL/AIC and rule of parsimony.

*NA= not able to be calculated

Abbreviations: PK=pharmacokinetic, -2LL=-2 Log-likelihood, AIC=Akaike information criterion, Imp=imprecision, CSF=cerebrospinal fluid, Tlag= lag term
